# Pituitary Stalk Enlargement With Panhypopituitarism and Arginine Vasopressin Deficiency in Lung Adenocarcinoma: Metastatic Infundibular Disease or Paraneoplastic Hypophysitis?

**DOI:** 10.7759/cureus.115095

**Published:** 2026-08-24

**Authors:** Rayhanul I Tuhin, Moieze R Malik, Joel Fernandez James, Cornelius Fernandez James

**Affiliations:** 1 Oncology, United Lincolnshire Hospitals NHS Trust, Boston, GBR; 2 Internal Medicine, Medical University of Sofia, Sofia, BGR; 3 Endocrinology, United Lincolnshire Hospitals NHS Trust, Boston, GBR

**Keywords:** adenocarcinoma lung, central diabetes insipidus (cdi), hypophysitis, panhypopituitarism, paraneoplastic syndrome

## Abstract

Paraneoplastic endocrine syndromes are well-recognised complications of lung malignancies, specifically small-cell lung cancer. The majority of patients with panhypopituitarism associated with lung cancer result from pituitary metastasis. In those with an enlarged pituitary stalk without obvious pituitary metastasis presenting with a combination of panhypopituitarism and arginine vasopressin deficiency (AVP-D), metastatic pituitary stalk infiltration should be considered rather than a paraneoplastic immune-mediated hypophysitis. We report a case of a 67-year-old gentleman with newly diagnosed lung adenocarcinoma, found to have panhypopituitarism before starting systemic anti-cancer treatment. Further hormonal tests revealed central hypothyroidism, hypogonadotropic hypogonadism and growth hormone deficiency. MRI of the pituitary demonstrated heterogeneous contrast enhancement without an obvious focal lesion. The pituitary stalk was slightly enlarged at the level of the optic chiasm, raising the possibility of hypophysitis. The patient was initiated on hydrocortisone and levothyroxine replacement therapy. Following hormone replacement, previously occult AVP-D became clinically apparent, manifesting as worsening dehydration, polyuria and polydipsia.

The overnight water deprivation test confirmed AVP-D. The patient showed subsequent clinical improvement with desmopressin. Serum prolactin was mildly elevated, likely reflecting both pituitary stalk infiltration and concomitant metoclopramide therapy. This case discusses an exceptionally rare presentation of lung adenocarcinoma with isolated pituitary stalk infiltration, panhypopituitarism, and AVP-D before exposure to immune checkpoint inhibitors. Clinicians should be aware of this unusual manifestation, as prompt hormone replacement can significantly reduce morbidity and improve patient outcomes.

## Introduction

Paraneoplastic syndromes are a group of clinical disorders that are secondary to tumour-associated hormonal secretion, cytokine release, or immune-mediated mechanisms without direct invasion by the primary tumour or metastatic deposits [1]. Roughly 10% of patients with lung cancer develop a paraneoplastic syndrome during the course of their disease [1].

Endocrine manifestations related to lung cancer include syndrome of inappropriate antidiuretic hormone secretion, humoral hypercalcaemia of malignancy due to parathyroid hormone-related protein, ectopic adrenocorticotropic hormone (ACTH) syndrome, carcinoid syndrome, and, less commonly, non-islet cell tumour hypoglycaemia and acromegaly resulting from ectopic secretion of insulin-like growth factors or growth hormone (GH)-releasing hormone [2-5].

Pituitary metastasis is well described with lung cancer [6,7]. In a case series and scoping review of 166 patients with pituitary metastases, 55 patients developed hypopituitarism, 41 developed arginine vasopressin deficiency (AVP-D), and 110 presented with visual symptoms. Lung cancer was the most common primary tumour (27.7%), followed by breast (18.7%) and kidney cancer (14.5%) [8]. 

Paraneoplastic autoimmune hypophysitis is an exceptionally rare immune-mediated endocrine syndrome, and presentation with panhypopituitarism in patients with lung cancer appears to be uncommon. Furthermore, involvement of both anterior and posterior pituitary function has been infrequently described in reported cases of paraneoplastic autoimmune hypophysitis [9-12]. Distinguishing paraneoplastic hypophysitis from metastatic infundibular disease is clinically important because management strategies differ substantially; paraneoplastic hypophysitis may warrant immunosuppressive therapy, whereas metastatic disease is managed primarily through treatment of the underlying malignancy together with appropriate hormone replacement.

We present a case of lung adenocarcinoma in which endocrine abnormalities were identified approximately 12 weeks after cancer diagnosis and before the initiation of systemic anti-cancer treatment. The patient developed panhypopituitarism and AVP-D associated with pituitary stalk enlargement. The case illustrates the diagnostic challenge of distinguishing paraneoplastic autoimmune hypophysitis from metastatic infundibular disease in a patient who had not received immune checkpoint inhibitor therapy. Although hypophysitis was initially considered because of pituitary stalk enlargement on MRI, the combination of AVP-D, combined anterior-posterior pituitary dysfunction, mild hyperprolactinaemia and heterogeneous pituitary enhancement ultimately favoured metastatic pituitary stalk infiltration as the most likely diagnosis.

## Case presentation

A 67-year-old male was referred for endocrine evaluation approximately 12 weeks after being diagnosed with stage IV lung adenocarcinoma. The referral followed abnormal biochemical investigations identified during routine screening before the initiation of systemic anti-cancer treatment. His Eastern Cooperative Oncology Group (ECOG) performance status was 2 at presentation. He reported progressive fatigue, lethargy, reduced exercise tolerance, weight loss, anorexia, and general malaise. There was no prior history of pituitary disease, autoimmune endocrinopathy, cranial irradiation, traumatic brain injury, or immune checkpoint inhibitor exposure. There were no headaches, visual disturbances or visual field defects.

Endocrine investigations demonstrated secondary adrenal insufficiency with very low 9 am serum cortisol levels on two occasions with suppressed plasma ACTH. The anterior pituitary hormone profile confirmed multiple deficiencies consistent with panhypopituitarism (Table 1).

**Table 1:** Endocrine investigations at presentation demonstrating anterior pituitary hormone deficiencies consistent with panhypopituitarism

Test	Result	Reference range
9 am serum cortisol (Day 1)	43 nmol/L	140-690
9 am serum cortisol (Day 2)	66 nmol/L	140-690
Adrenocorticotropic hormone (ACTH)	5 ng/L	7.2-63.3
Insulin-like growth factor 1 (IGF-1)	3.4 nmol/L	7.4-24.3
Prolactin	447 mIU/L	86-324
Follicle-stimulating hormone (FSH)	0.8 IU/L	1.5-12.4
Luteinising hormone (LH)	<0.3 IU/L	1.7-8.6
Testosterone	<0.1 nmol/L	6.7-25.7
Thyroid-stimulating hormone (TSH)	0.16 mIU/L	0.27-4.5
Free thyroxine (FT4)	4.0 pmol/L	11.0-23.0

**Figure 1:**
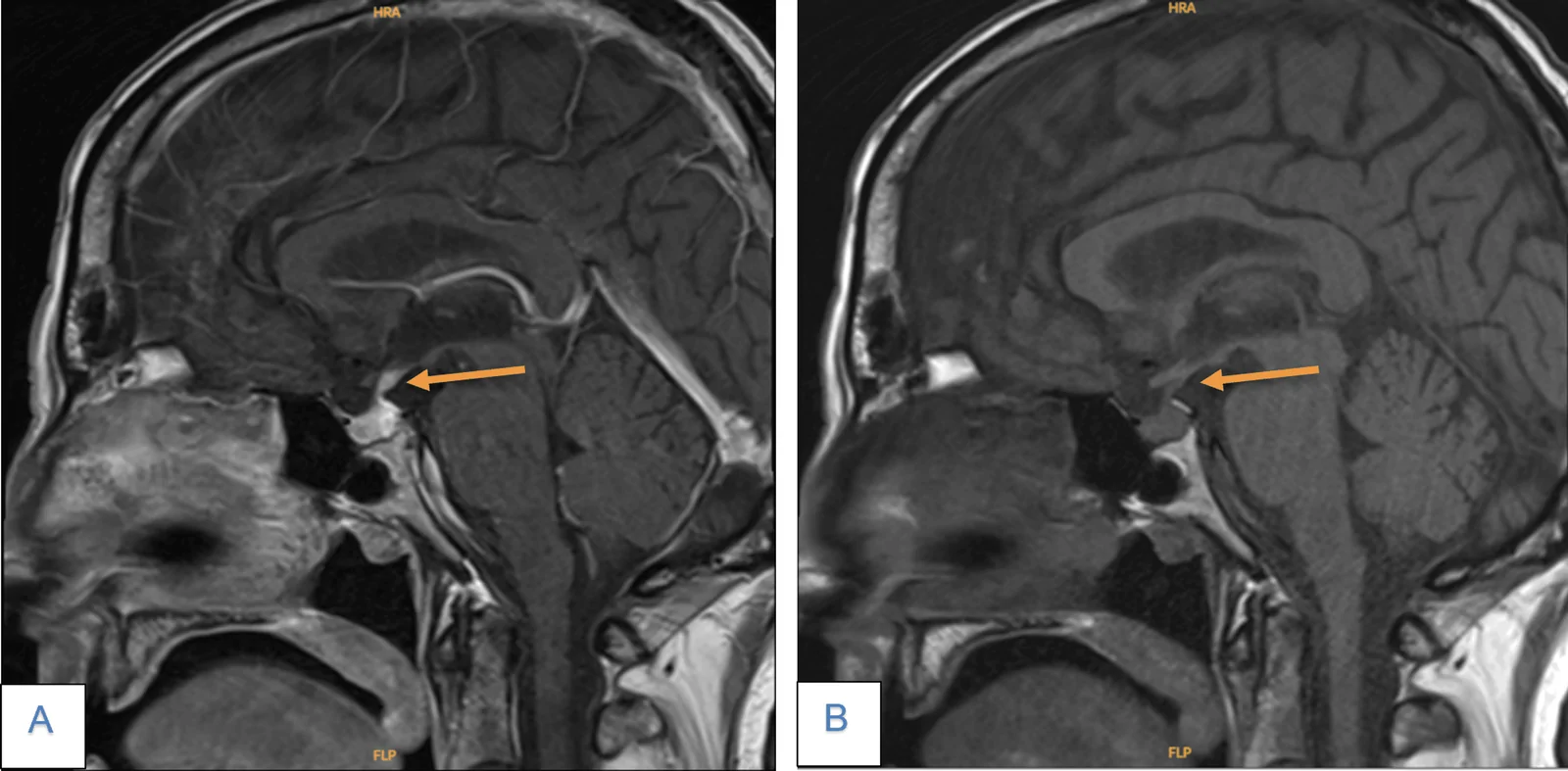
Sagittal magnetic resonance imaging (MRI) of the pituitary region (A) Post-contrast sagittal T1-weighted MRI demonstrating thickening and enhancement of the pituitary stalk. The orange arrow indicates the area of pituitary stalk thickening and enhancement. (B) Pre-contrast sagittal T1-weighted MRI of the pituitary region demonstrating the pituitary stalk prior to contrast administration. The orange arrow indicates the pituitary stalk.

MRI of the brain with dedicated pituitary imaging demonstrated heterogeneous contrast enhancement of the pituitary gland without evidence of an obvious focal lesion (Figure 1). The pituitary gland size and pituitary stalk diameter at the level of pituitary insertion were reported as normal. However, the pituitary stalk was noted to be slightly enlarged at the level of the optic chiasm. Although the radiological findings were subtle, the reporting neuroradiologist concluded that they may represent hypophysitis in the appropriate clinical and biochemical context. There was no radiological evidence of a pituitary adenoma or metastatic sellar lesion.

Hydrocortisone replacement therapy was commenced at a dose of 10 mg on waking, 5 mg at lunchtime, and 5 mg in the early evening. Levothyroxine 100 micrograms daily was subsequently initiated. The biochemical profile was consistent with central hypothyroidism, demonstrated by a low FT4 (4.0 pmol/L) in the presence of an inappropriately low thyroid-stimulating hormone (TSH) (0.16 mIU/L).

During his admission, he developed worsening malaise, persistent polyuria, excessive thirst and clinical dehydration despite consuming approximately 3-3.5 litres of water daily. Laboratory investigations revealed hypernatraemia, prompting further evaluation for possible AVP-D.

Fluid balance monitoring revealed a persistently negative fluid balance despite his significant oral intake. Given the combination of hypernatraemia, polyuria and polydipsia, endocrinology review raised suspicion for AVP-D. An overnight water deprivation test (approximately eight hours) was then carried out, which showed serum osmolality of 321 mOsm/kg and urine osmolality of 179 mOsm/kg. The findings of raised serum osmolality with low urine osmolality confirmed AVP-D. The patient was then started on intranasal desmopressin 100 micrograms, three times a day and showed subsequent symptomatic improvement.

The prolactin elevation was mild. Although metoclopramide therapy may have contributed to this, concomitant pituitary stalk involvement may also have resulted in a stalk effect. The modest degree of elevation was more consistent with stalk dysfunction than prolactin secreting adenoma, further supported by the absence of pituitary adenoma on the MRI.

During the subsequent course of his underlying malignancy, he developed severe sepsis. Despite appropriate medical management, his condition deteriorated, and he died secondary to sepsis of unknown source. Histological confirmation was unavailable because pituitary biopsy was considered high risk and unlikely to alter management. No post-mortem examination was performed. The diagnosis remains presumptive, although the overall clinical, biochemical and radiological findings supported the likelihood of metastatic infundibular disease. No systemic anti-cancer treatment had been commenced prior to death.

## Discussion

The salient features of the case include anterior pituitary dysfunction at presentation, unmasking of AVP-D following hydrocortisone and levothyroxine replacement, MRI findings of an enlarged pituitary stalk, and absence of a discrete pituitary mass or sellar metastatic lesion.

Pituitary metastasis is a recognised cause of hypopituitarism and panhypopituitarism in patients with malignancy. Patients commonly present with visual disturbance, headache, AVP-D (central diabetes insipidus), or varying degrees of anterior pituitary hormone deficiency, including panhypopituitarism [6,8]. Our patient demonstrated only subtle MRI abnormalities, which included heterogeneous pituitary enhancement and mild enlargement of the pituitary stalk at the level of the optic chiasm. MRI demonstrated subtle pituitary stalk enlargement without a discrete sellar metastasis. The MRI findings initially raised the possibility of hypophysitis; however, they were non-specific and could also have been explained by metastatic pituitary stalk infiltration. These findings broadened the differential diagnosis to include both autoimmune hypophysitis and metastatic infundibular disease.

The biochemical finding of hypernatraemia was pivotal to the diagnosis. While the patient initially complained of polyuria and polydipsia, the finding of hypernatraemia despite high oral fluid intake suggested impaired antidiuretic hormone (ADH) activity and prompted further investigation for diabetes insipidus. Water deprivation testing demonstrated raised serum osmolality with an inappropriately dilute urine (low urine osmolality), confirming AVP-D. The clinical response to desmopressin further supported the diagnosis.

The simultaneous finding of severe anterior pituitary hormone deficiencies with AVP-D demonstrated involvement of both pituitary compartments. Immune-mediated hypophysitis is most frequently reported as an adverse effect following treatment with immune checkpoint inhibitors, particularly anti-CTLA-4 agents [11]. However, this patient developed panhypopituitarism and AVP-D before receiving any form of immunotherapy, making treatment-induced hypophysitis unlikely.

An alternative explanation was a paraneoplastic immune-mediated hypophysitis triggered by the underlying cancer. In paraneoplastic autoimmune hypophysitis, ectopic expression of pituitary antigens within tumour tissue, including PIT-1 and, in some cases, pro-opiomelanocortin (POMC)-derived antigens, may trigger immune cross-reactivity against pituitary tissue, resulting in lymphocytic inflammation, pituitary stalk involvement and progressive hormonal dysfunction [12]. Similar autoimmune mechanisms have been proposed for several neurological and endocrine paraneoplastic syndromes associated with lung cancer [1].

However, paraneoplastic autoimmune hypophysitis is an exceptionally rare immune-mediated endocrine disorder. Currently, two major subtypes have been described: anti-PIT-1 hypophysitis, which targets somatotrophs, lactotrophs and thyrotrophs, resulting in GH, prolactin and TSH deficiencies, and anti-POMC hypophysitis, which targets corticotrophs and leads to secondary adrenal insufficiency [12]. To our knowledge, reported cases of paraneoplastic autoimmune hypophysitis have been limited to a small number of malignancies, including thymoma, diffuse large B-cell lymphoma, colon cancer, cholangiocarcinoma (anti-PIT-1 hypophysitis), and large-cell neuroendocrine carcinoma (anti-POMC hypophysitis) [12]. Reports associated with lung cancer appear to be extremely limited, particularly in patients presenting with combined panhypopituitarism and AVP-D prior to immune checkpoint inhibitor exposure.

In our patient with lung cancer presenting with panhypopituitarism and AVP-D before immunotherapy exposure, the combination of pituitary stalk enlargement, heterogeneous enhancement and combined anterior-posterior pituitary dysfunction raised suspicion for metastatic infundibular disease despite the absence of a discrete sellar metastasis.

The enlarged pituitary stalk and heterogeneous appearance of the pituitary gland raised the possibility of metastatic infundibular infiltration. In contrast, immune-mediated hypophysitis more typically demonstrates diffuse, symmetrical pituitary enlargement with pituitary stalk thickening and relatively homogeneous enhancement on MRI [13].

The posterior pituitary and infundibulum are the preferred sites for metastasis as they receive direct high-flow arterial supply from inferior hypophyseal arteries. On the other hand, the blood supply to the anterior pituitary comes from the low-pressure hypophyseal portal system [14]. Although pituitary metastases are uncommon, isolated pituitary stalk metastasis without a discrete sellar lesion appears particularly rare and has been reported predominantly in individual case reports and small case series [8,15-17].

Metastatic infiltration of the pituitary stalk may disrupt hypothalamo-pituitary signalling, impair portal blood flow, and interfere with axonal transport of AVP, resulting in anterior pituitary hormone deficiencies and central diabetes insipidus (AVP-D), including panhypopituitarism in severe cases [18]. The combination of panhypopituitarism and AVP-D in the presence of a relatively preserved pituitary gland raised suspicion for metastatic infundibular infiltration, a recognised manifestation of pituitary metastatic disease [18].

The emergence of polyuria following hydrocortisone replacement may reflect unmasking of previously occult central diabetes insipidus, a recognised phenomenon in patients with hypopituitarism [18]. The disruption of infundibular dopaminergic neurons or the hypophyseal portal blood flow that delivers dopamine (prolactin-inhibitory factor) to the lactotrophs could explain hyperprolactinemia in patients with pituitary stalk infiltration [19]. Cortisol deficiency increases ADH secretion and impairs free-water clearance, thereby masking diabetes insipidus; glucocorticoid replacement restores free-water clearance and unmasks the underlying vasopressin deficiency [20].

Distinguishing paraneoplastic hypophysitis from metastatic infundibular disease

The diagnostic challenge in this case was distinguishing paraneoplastic hypophysitis from metastatic infundibular disease. Features supporting hypophysitis included pituitary stalk enlargement, absence of a discrete sellar lesion, and the neuroradiological impression of hypophysitis. In contrast, the presence of untreated lung adenocarcinoma, AVP-D, combined anterior and posterior pituitary dysfunction, mild hyperprolactinaemia and heterogeneous enhancement favoured metastatic infundibular disease. A comparison of the distinguishing features is presented in Table 2. Histological confirmation was unavailable; metastatic infundibular disease was considered the more likely diagnosis.

**Table 2:** Comparison of paraneoplastic hypophysitis and metastatic infundibular disease Data summarised from the studies by Yang et al. [8], Bando et al. [9], Takahashi [10], Iglesias [12], and Komninos et al. [18]. AVP-D: Arginine vasopressin deficiency

Feature	Paraneoplastic hypophysitis	Metastatic infundibular disease	Present case
Underlying mechanism	Autoimmune inflammation triggered by tumour-associated antigens	Direct metastatic infiltration of the pituitary stalk/infundibulum	Both considered
Pituitary stalk thickening	Common	Common	Present
MRI appearance	Typically homogeneous pituitary enlargement with symmetrical stalk thickening	More commonly heterogeneous enhancement or focal stalk involvement	Heterogeneous enhancement with stalk enlargement
Anterior pituitary dysfunction	Common	Common	Panhypopituitarism present
AVP-D (central diabetes insipidus)	Uncommon	Common	Present
Hyperprolactinaemia	Variable	Common due to stalk effect	Mild elevation consistent with stalk dysfunction
Combined anterior and posterior pituitary dysfunction	Uncommon	Common	Present
Typical treatment	Hormone replacement ± immunosuppression	Hormone replacement with treatment of underlying malignancy	Hormone replacement provided
Diagnostic likelihood in this case	Less likely	More likely	Metastatic infundibular disease favoured

This case therefore represents a rare constellation of untreated lung adenocarcinoma, panhypopituitarism, hypernatraemia, AVP-D and pituitary stalk enlargement. Although hypophysitis was initially considered radiologically, the overall clinical, biochemical and imaging findings favoured metastatic infundibular infiltration as the most likely explanation.

## Conclusions

This case highlights the diagnostic challenge of distinguishing paraneoplastic hypophysitis from metastatic infundibular disease in untreated lung adenocarcinoma. Although pituitary stalk enlargement initially suggested hypophysitis, the presence of AVP-D, combined anterior and posterior pituitary dysfunction, mild hyperprolactinaemia and heterogeneous enhancement favoured metastatic pituitary stalk infiltration. Early recognition and hormone replacement are essential to reduce morbidity.
